# Lessons learnt from the first large outbreak of COVID-19 in health-care settings in Tasmania, Australia

**DOI:** 10.5365/wpsar.2021.12.4.884

**Published:** 2021-12-22

**Authors:** Fay H Johnston, Tara Anderson, Michelle Harlock, Natasha Castree, Louise Parry, Therese Marfori, Michelle McPherson, Mark Veitch, Kylie J Smith, Nicola Stephens

**Affiliations:** a Public Health Services, Department of Health, Tasmania, Australia.; b Menzies Institute for Medical Research, University of Tasmania, Tasmania, Australia.; c Tasmanian Health Service, Department of Health, Tasmania, Australia.; d Department of Health, Victoria, Australia.; e School of Medicine, University of Tasmania, Tasmania, Australia.

## Abstract

**Problem:**

One month after the initial case of coronavirus disease 2019 (COVID-19) in Tasmania, an island state of Australia, two health-care workers (HCWs) from a single regional hospital were notified to public health authorities following positive tests for SARS-CoV-2 nucleic acid. These were the first recognized cases in an outbreak that overwhelmed the hospital’s ability to function.

**Context:**

The outbreak originated from two index cases. Both had returned to Tasmania following travel on a cruise ship and required hospital admission for management of COVID-19. A total of 138 cases were subsequently linked to this outbreak: 81 HCWs (most being nurses) and 23 patients across three hospitals, one resident of an aged-care facility and 33 close contacts.

**Action:**

The outbreak was controlled through the identification and isolation of cases, identification and quarantining of close contacts and their household members, closure of the affected facilities and community-level restrictions to reduce social mixing in the affected region.

**Lessons learnt:**

Factors that were likely to have contributed to ongoing transmission in this setting included workplace practices that prevented adequate physical distancing, attending work while symptomatic, challenges in rapidly identifying contacts, mobility of staff and patients between facilities, and challenges in the implementation of infection control practices.

**Discussion:**

Many commonly accepted hospital practices before the COVID-19 pandemic amplified the outbreak. The lessons learnt from this investigation changed work practices for HCWs and led to wider public health interventions in the management of potential primary and secondary contacts.

## PROBLEM

**Figure 1:**
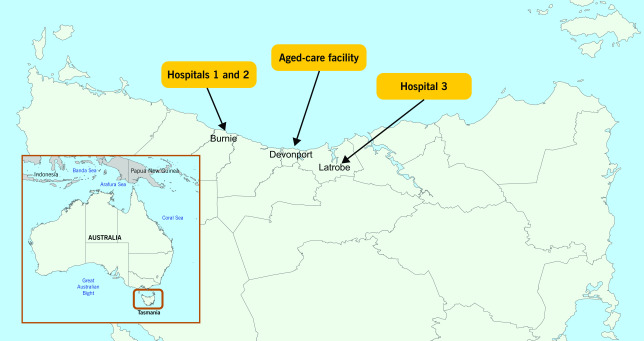
Map of Australia showing Tasmania (inset) and the northern coast of Tasmania showing the locations of the health-care facilities involved in the outbreak

On 19 March 2020, 2671 passengers and 1146 crew disembarked from a cruise ship after a 12-day international cruise that began and ended in Sydney, Australia; they then travelled on to other destinations. (*1*) Two thirds of the passengers were Australian; of these, 40% were subsequently diagnosed with coronavirus disease 2019 (COVID-19), including 18 who were diagnosed after returning to Tasmania, an island to the south of mainland Australia with a population of 528 000. Two of these Tasmanian cases were admitted to a regional public hospital on the north-western coast (Hospital 1) for management of their illness. Both were later identified as index cases of an outbreak that ultimately affected another 138 people comprising health-care workers (HCWs), patients and other close contacts. The outbreak led to the closure of Hospital 1; it also affected staff and patients at the co-located private hospital (Hospital 2), a smaller public hospital 56 km away (Hospital 3) and a residential aged-care facility 48 km away (**Fig. 1**). Here we describe the outbreak, possible transmission and lessons learnt from this early outbreak in Australia.

[**insert** Figure 1]

## CONTEXT

The Tasmanian outbreak was the first large COVID-19 outbreak to occur in Australia within a health-care setting that demonstrated ongoing transmission between HCWs. Tasmania’s initial COVID-19 case was notified on  2 March 2020, and there was no community transmission in Tasmania at that time. HCWs are at risk of acquiring COVID-19 infection from their patients and of subsequently instigating or amplifying outbreaks within the health-care setting. (*2*, *3*) In recognition of the anticipated increased risk posed by the pandemic, hospitals in Tasmania had strengthened infection prevention and control procedures even though, before this outbreak, only nine patients with COVID-19 had been managed in a hospital in Tasmania.

### Description of outbreak

Outbreak cases were defined in accordance with Australian national guidelines (*4*) as persons with laboratory confirmation of COVID-19 by nucleic acid testing from a deep nasopharyngeal swab, with onset of illness on or after 19 March 2020, who had a direct or indirect epidemiological link to any of the three health-care facilities (Hospitals 1–3) in the north-western region of Tasmania. All laboratory-confirmed cases were notified to Public Health Services (PHS), Tasmanian Department of Health, for public health response, as required by legislation. Cases were contacted to collect information about age, sex, occupation and risk factors for acquisition of infection, and to identify close contacts, as defined by the national guidelines. (*4*) Employment records were used to determine the number of staff by occupational group at Hospital 1 to estimate attack rates among clinical occupational groups.

**Figure 2:**
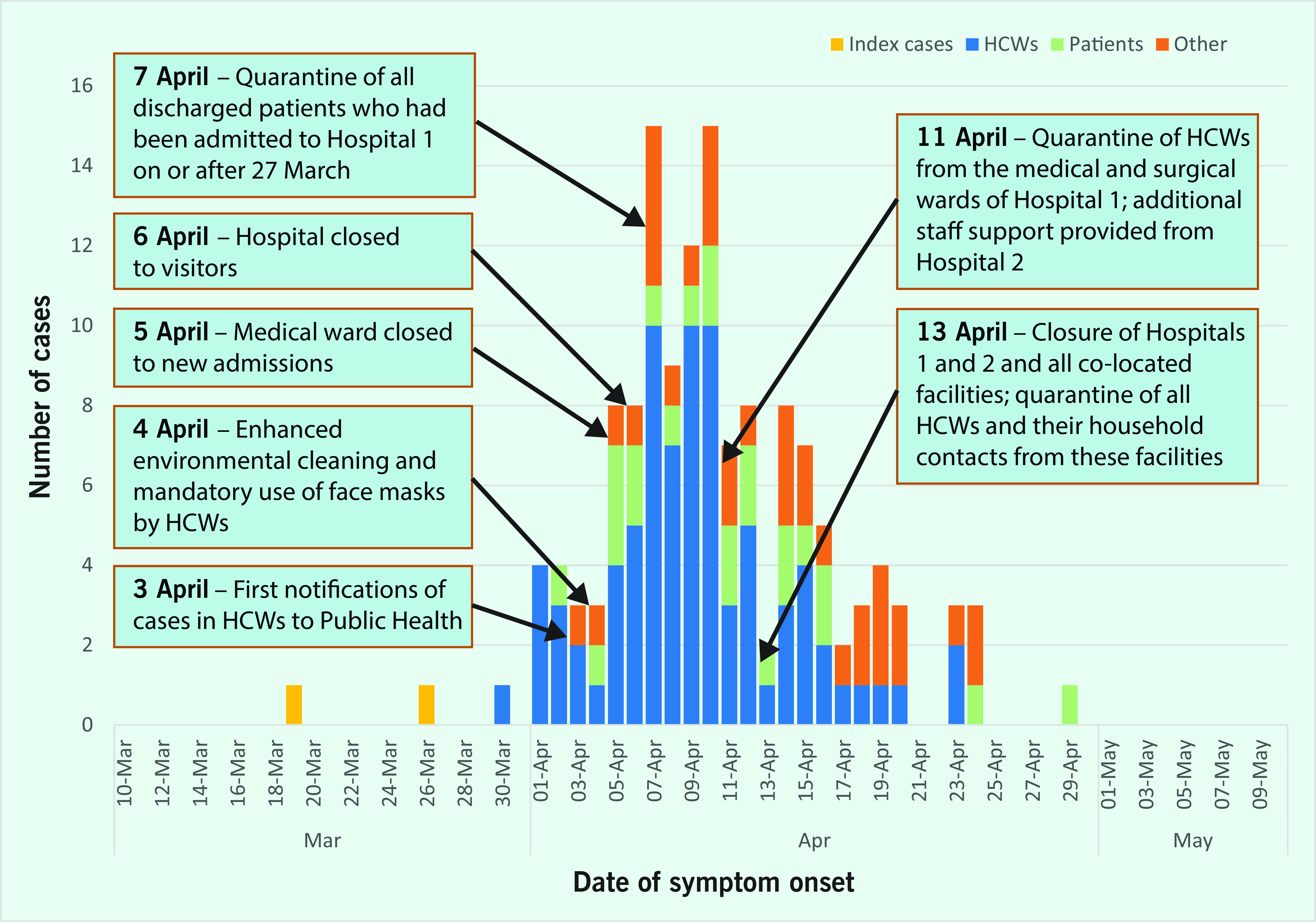
MEpidemic curve of COVID-19 cases associated with the northwest outbreak in Tasmania, Australia, March to May 2020

The two index cases were admitted to the medical ward of Hospital 1 for the management of COVID-19 on 20 and 26 March 2020. Their respective dates of diagnosis and notification to the Department of Health were 19 and 26 March. The two initial cases in HCWs were notified on 3 April 2020, with a third HCW case notified the following day. All three HCW cases worked on the medical ward of Hospital 1, although none provided direct care to the two index patients. Thereafter, daily COVID-19 case numbers increased rapidly for 10 days before declining (**Fig. 2**).

[**insert** Figure 2]

A total of 138 cases and 10 deaths were linked to the outbreak. Of the cases, 81 were HCWs, 23 were patients across the three hospitals, one was a resident of the aged-care facility and 33 were close contacts. The close contact cases included a small community cluster of six cases initiated from a discharged patient. The age and sex distributions of cases are shown in **Table 1**.

**Table 1:** Age and sex distribution of cases by group, hospitalization and death

-		All	Health-care workers	Patients^a^	Other^b^	Hospital cases^c^	Deaths
Total	-	138	81	24	33	29	10
Sex	Female	85	61	7	17	12	5
	Male	53	20	17	16	17	5
Age group (years)	0–19	6	–	–	6	–	–
	20–49	67	52	1	14	2	–
	50–69	41	28	5	8	9	–
	70+	24	1	18	5	18	10

^a^ Includes people being treated in hospital or residents in an aged-care facility.

^b^ Includes household and other close contacts of people with COVID-19 infection.

^c^ Includes people who acquired the illness as inpatients and those who acquired the illness out of hospital but required admission for treatment of COVID-19.

### Cases among HCWs

Of the 81 cases among HCWs, 72 (89%) worked within Hospital 1, some of whom also worked at other facilities during the outbreak period, and 49 (60%) were nurses. Cases also occurred among medical and allied health practitioners, and among people working in maintenance, administrative and catering services, but none were identified among cleaning staff. The attack rates at Hospital 1 were 16/98 doctors (16%) and 43/393 nurses (11%). Seven HCWs required admission to hospital for management of their illness and all were subsequently discharged.

Affected HCWs worked across facilities in the co-located medical precinct of Hospitals 1 and 2 (including in pathology collection and outpatient services) and in health-care facilities in other locations. The median number of different clinical settings where individual staff worked during their infectious period was 1 (range 1–7). A total of 40 (49%) HCW cases did not attend work while symptomatic, 26 (32%) first had symptoms on their last day at work and 15 (19%) attended work while symptomatic for time periods of 1–7 days. Seven asymptomatic cases were identified during the outbreak, mostly through the requirement for testing before resuming work when Hospital 1 was reopened.

### Pathways of transmission

The initial cases notified to PHS occurred in staff primarily working on the medical ward of Hospital 1. Ten HCWs had onset of symptoms between 30 March and 3 April, before identification of the first HCW cases, and at least two of these HCWs recalled providing direct care to one of the two index cases during their acquisition period. These early cases included medical, nursing and allied health staff who attended daily nursing and medical handover meetings conducted in confined spaces. Several other clusters among HCWs were identified among attendees of regular meetings, such as administrative or clinical planning meetings.

Cases also occurred in the co-located Hospital 2 (9 HCWs and 6 patients); among these cases, six (5 HCWs and 1 patient) had no link to other health-care facilities. Fourteen cases were associated with Hospital 3 (4 HCWs and 10 patients), of whom three (2 HCWs and 1 patient) could only have acquired the infection at Hospital 3, whereas the remainder had either worked at or had also been admitted to Hospital 1. The single case from the residential aged-care facility acquired COVID-19 from a HCW who had previously worked at both Hospitals 1 and 2.

## ACTION

A description of the management of the outbreak has been published elsewhere, (*5*) and key elements are summarized here. Following the initial notifications, emergency response teams were established at Hospital 1 to identify and quarantine close contacts of cases and manage the outbreak consequences in the hospital. Concurrently, the Public Health Emergency Operations Centre (PHEOC) increased its workforce of contact-tracing personnel, public health nursing and medical staff, and epidemiologists, to manage the escalating numbers of cases and contacts requiring investigation. Staff were sourced through government interoperability arrangements and secondment agreements with the University of Tasmania.

Initial interventions at the hospitals included enhanced environmental cleaning, use of surgical face masks by all HCWs in the medical and surgical wards in Hospital 1, and prohibition of visitors to Hospitals 1 and 2. Interventions escalated rapidly as case numbers continued to increase. On 7 April 2020, admission of new patients to the medical and surgical wards of Hospital 1 ceased, and external specialist support was increased, including an infectious disease physician and a mobile PHEOC team comprising a public health physician, an epidemiologist and a clinical nurse consultant. On 10 April, all remaining HCWs from the medical and surgical wards, who had not already been identified as close contacts, were placed in quarantine.

By 12 April, cases had been identified across most clinical areas of Hospital 1 (including medical, surgical and mental health wards, and operating theatres), Hospital 2, and in the pathology service and outpatient clinics co-located with these facilities. On 13 April, Hospitals 1 and 2 and related campus medical services were closed, with patients transferred to other facilities, including Hospital 3. All HCWs who had worked in Hospitals 1 and 2 and co-located facilities from 27 March (approximately 1300 people) and their household members (an estimated additional 3000–4000 people) were placed in quarantine at home for 14 days. This was the first example of the quarantining of secondary close contacts for outbreak management in Australia.

Community restrictions were also implemented on 12 April to reduce social mixing in the affected region. This included a 14-day closure of all non-essential retail businesses, the strictest restrictions in Australia at the time. (*5*) The Australian Defence Force provided temporary emergency department services while Hospital 1 was cleaned, recommissioned and reopened.

These control measures were followed by a reduction in the number of new cases over the following days. The outbreak was declared over on 6 June, after two incubation periods (i.e. 28 days) had passed with no new cases.

## LESSONS LEARNT

We identified several factors that contributed to and amplified the spread of COVID-19 through the health-care settings.

### Physical distancing

The nature of clinical work in a hospital makes it difficult to maintain physical distancing between staff, and between patients and staff. Studies have found no difference in seroprevalence rates between frontline and non-frontline staff, highlighting transmission routes outside of direct patient care, such as from staff to staff. (*6*, *7*) These factors were illustrated in this outbreak by the clustering of cases among attendees of recurring events such as nursing handovers and discharge planning meetings. (*5*) The higher attack rates in doctors at Hospital 1 might be attributable to the sharing of offices, daily visits to most hospital wards, ward rounds in small groups that huddle around a computer screen and attendance at meetings. Hospital meeting places are often small, and cumulative time of close physical contact increases the risk of transmission. (*8*, *9*) Several measures, including limits on the number of people in rooms, were introduced after the outbreak to address physical distancing, although space constraints mean that assigning individual office space is often not possible.

### Presenteeism

Almost 20% of infected HCWs worked while symptomatic, with more unknowingly working during the pre-symptomatic stage of illness, an important infectious stage of COVID-19. (*9*) Some, especially those with pre-existing chronic respiratory conditions, attributed mild symptoms to other causes and were unable to differentiate such symptoms from the onset of COVID-19. It is also possible that some had asymptomatic COVID-19 infection. However, at this stage of the pandemic, the importance of asymptomatic infection and transmission had not been recognized; hence, testing of asymptomatic contacts was not standard practice. (*4*, *10*)

Changing work practices relating to presenteeism (i.e. attending work when unwell) requires a cultural shift in long-standing attitudes and perceptions that increase the likelihood of this behaviour. Reasons for individuals continuing to work include workplace culture and expectations, a desire to support their colleagues, especially when there are staff shortages, and to maintain income, a particularly important consideration for casual workers. (*11*, *12*) Interventions were subsequently introduced to support this cultural change, including screening staff for acute respiratory symptoms before each shift, requiring COVID-19 testing for staff who develop acute respiratory infection, and developing operational frameworks to support staff absences due to symptomatic respiratory infections and while awaiting test results.

### Contact identification and testing

There were many challenges with the timely identification of close contacts from the three hospitals. One challenge was locating multiple electronic and paper-based information systems to identify staff and patient movements during the outbreak, often by outbreak investigation team members unfamiliar with the local setting. COVID-19 response guidelines and the definition of a close contact were frequently updated throughout the investigation and, as the outbreak escalated, contact tracing became overwhelming for the number of contact tracers available. (*5*) These logistical difficulties made quarantining close contacts challenging. (*9*, *12*)

The outbreak occurred early in the pandemic when national guidelines limited COVID-19 testing to symptomatic individuals and access to rapid testing was limited. (*4*) Consequently, not all contacts were tested. This hindered the rapid identification of new cases and may have resulted in asymptomatic cases going undetected, potentially adding to transmission. Outbreak management principles, including the testing of asymptomatic contacts, were later added to the Australian series of national guidelines for COVID-19 on 28 May 2020. (*4*)

### Staff and patient mobility

Many infectious staff were highly mobile within the health-care facilities or worked in more than one  health-care setting. Given the small regional hospital workforce in this location, the mobility between  health- care and aged-care facilities was unavoidable. Several patients who were transferred between hospitals were infectious but had not yet been diagnosed with COVID-19; this contributed to transmission from Hospital 1 to Hospitals 2 and 3 early in the outbreak.

### Infection prevention and control practices

Independent transmission from the two index cases with COVID-19 to HCWs was confirmed by genomic analysis, (*13*) and a later instance of transmission from a COVID-19 patient to a HCW at Hospital 3 was identified through epidemiological investigation. (*5*) Although no specific breaches of infection control protocols were recalled by the HCWs concerned, strengthening of infection control practices for all HCWs through increased resourcing and staff education, training and support was rapidly implemented following the outbreak. (*12*)

## Discussion

Despite no ongoing community transmission in the region, the Tasmanian outbreak was characterized by rapid transmission in health-care settings, with staff-to-staff transmission as the most significant contributor to the escalation of cases. The investigation identified a range of existing HCW practices that facilitate disease transmission in hospital settings, including challenges in achieving physical distancing, a culture of presenteeism and a high level of mobility of staff and patients across multiple health-care settings. The rapid closure of two hospitals highlighted the difficulties of maintaining a workforce in rural settings, because increases in demand coincided with a diminishing workforce due to the escalating isolation and quarantine requirements. (*14*)

The requirement that all HCWs from Hospitals 1 and 2 and their household contacts quarantine for 14 days, regardless of whether they met existing definitions of a close or casual contact, was associated with achieving rapid control of the outbreak. The definitions of close, casual and secondary contacts have since been refined, and criteria for quarantine of people in these groups form part of the current series of national guidelines for COVID-19. (*4*)

A limitation of the study is the lack of information about asymptomatic cases. At the time of the outbreak, the availability of rapid testing was limited, and testing of asymptomatic contacts was not routinely conducted. Although seven (5%) of the known 138 cases were found to be asymptomatic, this could be an underestimate. It has been estimated that up to 24% of transmission could be associated with asymptomatic disease. (*15*)

The learnings from this first large Australian outbreak in a health-care setting have contributed to ongoing interventions and pandemic responses throughout Tasmania and other states and territories of Australia.
